# An Optical Chiral Sensor Based on Weak Measurement for the Real-Time Monitoring of Sucrose Hydrolysis

**DOI:** 10.3390/s21031003

**Published:** 2021-02-02

**Authors:** Dongmei Li, Chaofan Weng, Yi Ruan, Kan Li, Guoan Cai, Chenyao Song, Qiang Lin

**Affiliations:** Key Laboratory of Quantum Precision Measurement of Zhejiang Province, Center for Optics & Optoelectronics Research, Collaborative Innovation Center for Information Technology in Biological and Medical Physics, College of Science, Zhejiang University of Technology, Hangzhou 310023, China; lidm18@zjut.edu.cn (D.L.); cfweng1022@gmail.com (C.W.); yiruan@zjut.edu.cn (Y.R.); kanli@zjut.edu.cn (K.L.); caiguoan21@gmail.com (G.C.); chenyaosong302@gmail.com (C.S.)

**Keywords:** chiral sensor, optical sensors, weak measurement, sensor systems and applications, sucrose hydrolysis

## Abstract

A chiral sensor with optical rotation detection based on weak measurement for the kinetic study of sucrose hydrolysis is presented. Based on the polarization modulation to the pre-selection state, the optical rotation of chiral sample was accurately determined through the central wavelength shift of the output spectrum. With this approach, the concentration response curves of sucrose and its hydrolysis products, i.e., fructose and glucose, were experimentally obtained for the hydrolysis analysis. By collecting the output spectrum with a frequency of 100 Hz and fitting the central wavelength shift synchronously during the measurement, the sucrose hydrolysis process was monitored in real time. Different hydrolysis conditions with varied concentration of invertase enzyme and citrate were implemented in this work. As a consequence, the real-time hydrolysis curves of the hydrolysis process with distinct velocities was achieved and analyzed. Such a kinetic monitoring about sucrose hydrolysis with optical rotation detection technology played a critical role in the researches involving sucrose, and also revealed the great potential of weak measurement in intersections, such as food safety inspection and chemical analysis.

## 1. Introduction

As the main carbohydrate reserve and energy source in human diet, sucrose exists extensively in plants. However, the functional properties of sucrose are limited as a polysaccharide, which is difficult to be metabolized and absorbed directly. The hydrolysis process of sucrose produces an equimolar monosaccharide mixture, i.e., glucose and fructose. Such a transformation provides a sweetener, which is desirable in food and medicine industries with the advantages of an instant energy source, high osmotic pressure and low crystallization [1]. In addition, the roles of invert syrups in food industry are also reflected in the manufacturing of soft-centred candies and fondants as a humectant [2]. Hence, numerous efforts have been made to optimize hydrolysis process, and varied catalytic methods [3,4] were utilized to improve the hydrolysis efficiency. As a fundamental tool for the monitoring of hydrolysis process, a kinetic measurement technology is significant for the studies involving sucrose hydrolysis. Rapid analytical methods, such as nuclear magnetic resonance (NMR) spectroscopy [5,6] and in situ FTIR spectroscopy [7], have been reported for the kinetic and mechanistic study of sucrose hydrolysis. Besides, the kinetics of sucrose hydrolysis was also monitored with chemical reactors [8,9]. However, the real-time response curve of the hydrolysis reaction, which is a fundamental tool for the understanding and controlling of the chemical processes, has not yet implemented. Besides, the heavy equipment and complex operations are the main limitations of these approaches for universal application in industry. The real-time monitoring of the sucrose hydrolysis with high-sensitivity, high-quality, easy integration and up-to-date equipment is still required in food science and industrial production.

Optical technology shows distinct superiority in real-time sensing in numerous applications, such as chemical sensing and biosensing [10,11,12]. Especially, optical sensors based on surface plasmon resonance (SPR) have played a non-negligible role in the area of chemical sensing, foodborne marker screening, environmental monitoring and medical diagnostics [13]. They were also utilized for the detection of sucrose [14,15], which was taken as an important refractive index of medium to verify sensor performance. However, with the feature of refractive index dependence, which may cause undesirable reference in the kinetic study of sucrose hydrolysis, SPR sensors have not yet been exploited in the real-time monitoring of sucrose hydrolysis. As one important feature of sucrose and its hydrolysis productions, which are all chiral materials, optical rotation is a crucial breakthrough for the analysis of sucrose hydrolysis process, and it shows a unique merit in the rapid analysis of optical activity compared with traditional chiral sensing technologies. Even so, optical rotation detection was rarely reported to be applied in the studies on the monitoring of sucrose hydrolysis or other chiral reactions due to the limitation of precision.

Recently, quantum weak measurement scheme in optical system, which exhibits a great potential in high precision detection of physics parameters [16,17,18], has been introduced in interdisciplinary researches [19,20,21]. And it was also reported to be potential in real-time monitoring of biomedical and chemical reaction [22,23]. In 2011, Marcel Pfeifer and Peer Fischer discussed how quantum weak measurement can be adapted to measure optical activity. They utilized the refraction geometry at a chiral-achiral interface for the optical activity measurement with weak value amplification and realized a sensitivity of 0.00003° solutions in ∼µL volumes [24]. Such a sensitivity indicated a significant evaluation of optical detection technology for optical rotation detection. Subsequently, Zhang and his colleagues reported the scheme of weak measurement for optical rotation detection based on spin Hall effect of light (SHEL) [25]. Especially, they investigated ultrasensitive and real-time detection of chemical reaction rate based on the photonic spin Hall effects on the basis of sucrose hydrolysis [26]. However, a real-time response curve of the reaction, which could reveal the hydrolysis efficiency on line, was still desired.

In this work, we built a weak measurement system based on a super luminescent diode (SLD) with a wide band in a common-path. In this system, the polarization angle of pre-selection could be determined via the central wavelength shift of output spectra. In this system, optical rotation of the chiral sample could be evaluated sensitively with a resolution of $2.138\times 10^{-5{}^{\circ}}$ through spectrum analysis [27]. Meanwhile, differ from SPR sensors, this weak measurement system has a unique merit in real-time sensing because of its refractive index independence [28]. Based on it, by fitting the central wavelength shift with the frequency of 100 Hz, we obtained a kinetic response curve for the varied optical activity of the sample. Compared with existing technologies, such as traditional polarimeter, which is a standard technology for optical rotation detection, this chiral sensor based on weak measurement showed an advantage of high precision [27], and it did realize the function of real-time monitoring. With the high sensitivity on-line monitoring of the hydrolysis process, the hydrolysis degree could be observed intuitively and the optimal hydrolysis conditions could be adjusted reasonably. Moreover, this chiral sensor based on weak measurement was realized with a 830 nm light source. This near-infrared light source with the wavelength in 700–900 nm has a low absorption in water and hemoglobin, and can propagate as deep as 15 cm in biological tissue [29]. Hence, it may have a great potential in the on-line detection of chiral molecule, such as blood glucose, in living tissue. Such a demand cannot be met by existing optical rotation sensors, for example, current commercial polarimeters all work with the light source of visible wavelength. Besides, this optical chiral sensor for sucrose hydrolysis monitoring displays the superiority of easy integration, which is a crucial factor for the universal applications in various areas.

## 2. Materials and Methods

### 2.1. Optical Rotation Detection Based on Weak Measurement in Frequency Domain

**Figure 1:**
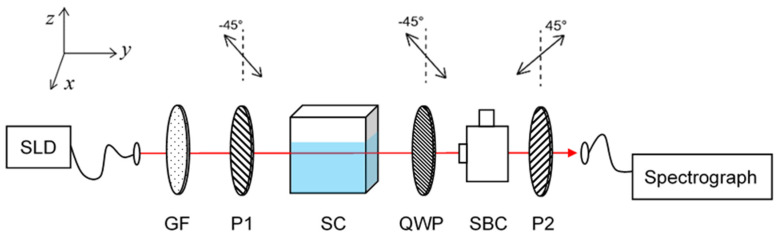
The experimental setup of optical weak measurement system. SLD, super luminescent diode. GF, Gaussian filter. P1 and P2 represent two same polarizer. SC, sample cell, especially for the holding of the liquid sample in detection. SBC, Soleil-Babinet compensator. QWP, quarter wave plate.

The optical system based on weak measurement for optical rotation detection was built as shown in Figure 1.

In this system, the light source SLD (SLD830S-A20, Thorlabs Inc., Newton, NJ, USA) centered at 830 nm with a bandwidth of 20 nm. The light beam from SLD propagated along the y direction. A GF was utilized to modify the spectral profile with a Gaussian peak, which is necessary for the weak measurement in frequency domain. P1, QWP and P2 were located respectively with the angle of −π/4, −π/4 and π/4 versus the vertical direction. As described in Ref. [27], the pre-selected polarization was realized with the combination of P1, SC and QWP. It can be expressed as Equation (1) while α  represented the angle change induced by P1 or the optical activity of the sample in SC:

(1) $$|\psi_{i}=\frac{\sqrt{2}}{2}[-e^{i\alpha }|H\rangle +ie^{-i\alpha }|V\rangle ]$$

SBC and P2 contributed to the establishment of the post-selected polarization state as expressed as Equation (2):

(2) $$|\psi_{f}=-\frac{\sqrt{2}}{2}[-|H\rangle +e^{i\beta }|V\rangle ]$$

Here, β  represents the phase difference between |H〉 and |V〉 induced by SBC. The quantitative relation between the wavelength shift and the phase difference as well as the polarization angle can be displayed as Equation (3), which was deduced in previous publication [27] in detail:

(3) $$\delta \lambda =-\frac{4\pi {\left(\Delta \lambda \right)}^{2}}{\lambda_{0}}ImA_{w}=-\frac{2\pi {\left(\Delta \lambda \right)}^{2}\tan\left(\frac{\pi }{4}-\frac{\beta }{2}-\alpha \right)}{\lambda_{0}}$$

Here,$\;A_{w}$ is the weak value of this weak measurement system, defined as $A_{w}=\langle \psi_{f}\left|A\right|\psi_{i}\rangle /\langle \psi_{f}|\psi_{i}\rangle$ with the observe operation A=[|V〉⟨V|−|H〉⟨H|]/2. $\lambda_{0}$ is the initial central wavelength 830 nm, and the bandwidth Δλ is 10 nm provided by the GF. Thus, with the relationship between the wavelength shift δλ and the angle, the optical rotation could be determined by the spectrum analysis in frequency domain. In this system, a spectrograph is connected to the computer, with which the collected spectra will be further processed by a LabVIEW program (National Instruments Corp., Austin, TX, USA).

**Figure 2:**
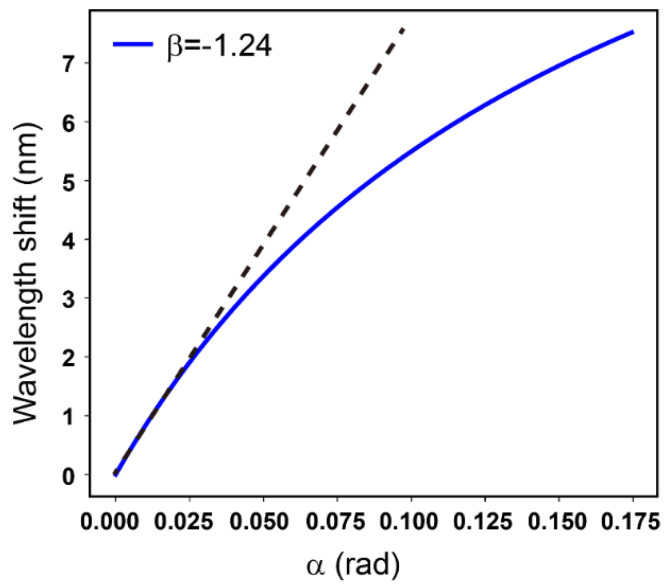
The theoretical response curve of wavelength shift with respect to α. The dotted line reveals the linear range for α detection.

In this work, the value of β was set to −1.24 rad. Hence, according to Equation (3), the central wavelength shift δλ, which was represented with wavelength shift in all figures in this paper, was fitted theoretically with respect to the optical rotation angle α, as shown in Figure 2.

As Figure 2 shows, the wavelength shift increased lineally with the change of α from 0 to 0.02 rad, which is appropriable in optical rotation detection for chiral molecule. In this linear range, the sensitivity, which could be expressed by the slope of the curve, was calculated to be 87.6 nm/rad. Compared with the sensitivity of 209.17 nm/rad reported in Ref. [27], the resolution for optical rotation should be $5.21\times 10^{-5{}^{\circ}}$.

### 2.2. Sucrose Hydrolysis

**Figure 3:**
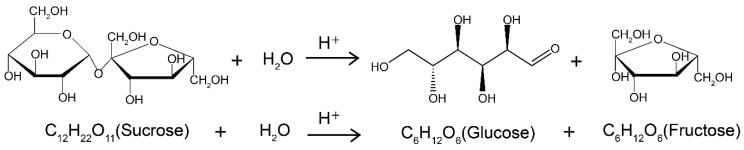
The chemical formula of sucrose hydrolysis.

Sucrose hydrolysis process was performed according to the chemical formula shown in Figure 3. In the action of a molecule of water, a sucrose (C12H22O11) molecule converted to be a molecule of glucose and fructose, which could be expressed with the same formula (C6H12O6).

### 2.3. Materials

The reagents in this work includes sucrose, glucose and fructose. Sucrose and glucose were purchased from Aladdin (Shanghai, CHN), with an analytical grade of ≥99.5% (GC). The citrate of analytical grade was also purchased from Aladdin. While the other two with a purity of 99% were provided by Shanghai Yuanye Bio-Technology Co., Ltd. (Shanghai, CHN).

## 3. Results and Discussion

### 3.1. Polarization Response of Weak Measurement System

In the weak measurement system, the output probe will be reshaped with the action of post selection state. In the frequency domain, the spectrum of the light always plays the role of a probe, and would be reshaped by the phase or polarization change in the measurement. According to Equation (3), the wavelength shift δλ of output spectrum is determined by the polarization angle α  of the preselection state.

**Figure 4:**
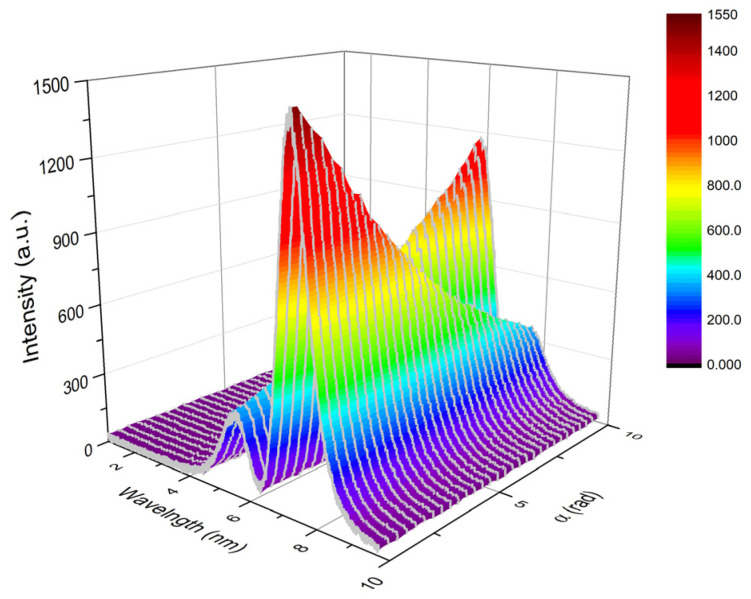
The output spectra of the weak measurement system with varied α angle.

In the system displayed in Figure 1, the α angle was adjusted by rotating P1 with an internal of 0.4′. The output spectra were collected and exhibited in Figure 4. As discussed in Ref. [30], the system was prepared in an optimal state, which has a spectrum with two-peaks. With an increasing α angle, the right peak of the spectrum was falling, and the left peak raised accordingly. That is, the central wavelength, which was achieved by fitting each spectrum for the center of gravity, shifted in the direction of decreasing. Hence, by fitting the spectrum for the central wavelength, the rotation angle of the polarization can be estimated with the wavelength shift.

### 3.2. Experiments for Optical Rotation Detection

**Figure 5:**
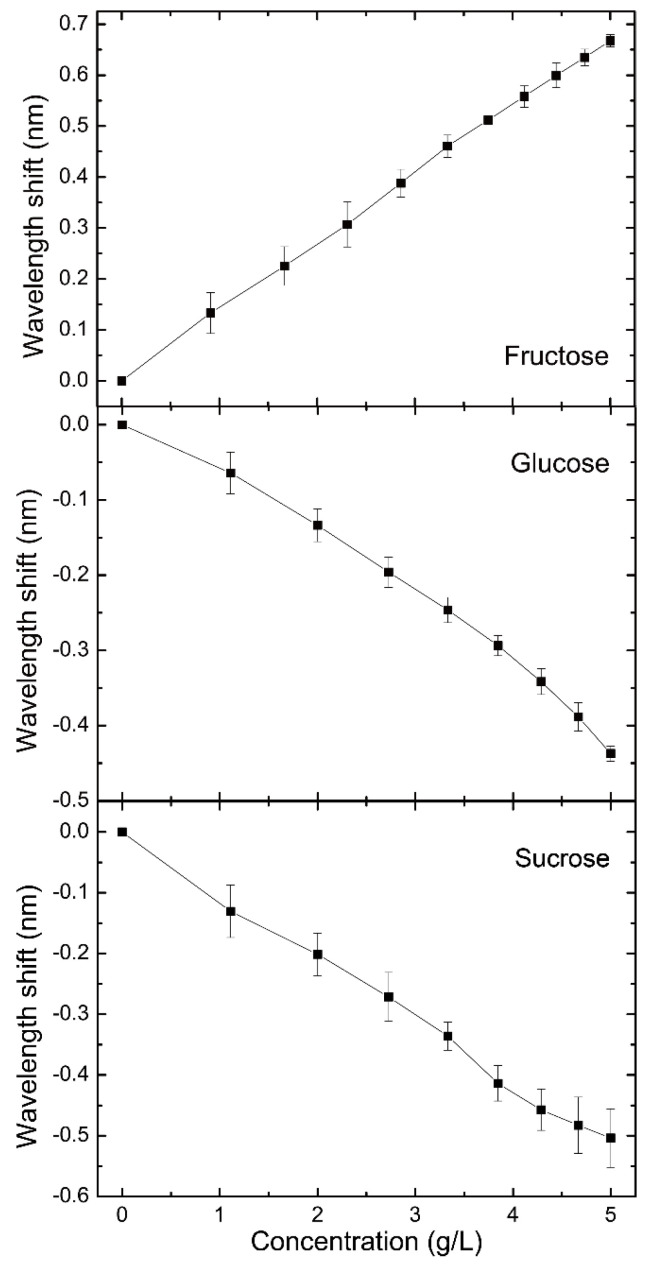
The concentration response curves of fructose, glucose and sucrose.

In this section, the solution of sucrose, glucose and fructose were detected with this system based on weak measurement. The chiral solution in SC with an optical length of 10 cm provided a polarization modification for the pre-selection state, i.e., the optical rotation angle α. For the determination of α, the initial sugar solution was prepared with the concentration of 10 g/L. In the measurement, the deionized water in SC was utilized to ensure the light beam propagated under the surface of the sample. Then, the prepared sugar solution with a volume of 1 mL was added into SC regularly. Slight stirring accelerated the diffusion of sugar molecules. The output spectrum was collected and fitted for the central wavelength. Compared with the initial parameter of 830 nm, the wavelength shift was obtained. Combining with the concentration calculation as for the solution after each sample adding, we achieved the concentration response versus wavelength shift as shown in Figure 5.

Figure 5 indicates the response relation of these three sugars between the concentration and the spectrum signal. Because of the optical activity, the specific optical rotation α was estimated with the weak measurement scheme by modifying the pre-selection polarization of the system. In the circumstance of 20 °C, the specific optical rotation of sucrose is known to be 66.6°, whereas that of glucose and fructose is 52.2° and −92.4°, respectively. Hence, with the increasing concentration, the wavelength shift performed a positive growth for fructose. Conversely, it had a negative growth for glucose and sucrose as a result of the contrary optical activity. Additionally, the magnitude of the wavelength shift for these three measurements display a clear distinction.

The rotation angle of chiral solution, i.e., α, can be calculated with the specific optical rotation of [*α*], as shown in Equation (4):

(4) $$\alpha =\frac{\left[\alpha \right]\cdot l\cdot c}{100}$$

In this equation, [*α*] is the specific rotation of chiral sample, *l* is the interaction length that was 1 dm in current system, and C is the concentration of chiral sample with a unit of g/100 mL. According to the theoretical fitting in Figure 2 and Equation (4), the optical rotation changing from 0 to 0.02 rad, which was corresponding to the upper concentration limitation of 17.21 g/L, 21.95 g/L and 12.40 g/L, could guarantee the linear range of the measurement with the β of −1.24 rad. The resolution of the system was provided as $\;5.21\times 10^{-5{}^{\circ}}$, which signified a low limitation of $7.82\times 10^{-4}\;$ g/L, $9.98\times 10^{-4}\;$ g/L and $5.64\times 10^{-4}\;$ g/L for the detection of sucrose, glucose and fructose, respectively.

### 3.3. Discussion of the Repeatability and Reliability in Real-Time Monitoring of Sucrose Hydrolysis

For this weak measurement system, the integral time of the spectrograph was 10 ms, that is, the sampling rate is 100 Hz. In the measurement, the central wavelength shift of output spectrum could be fitted in real time with the Labview programming. Hence, the polarization angle, induced by the first polarimeter or the optical rotation of the chiral sample, could be monitored in real time. In this work, sucrose hydrolysis was implemented in the SC of the weak measurement system. The invertase enzyme was exploited to speed up the reaction. Firstly, the sucrose solution was prepared with a concentration of 10 g/L, and 12 mL of the sucrose was added in the SC. Then, invertase enzyme was mixed in the sucrose solution. The central wavelength of output spectrum was collected synchronously. Compared with the initial wavelength, i.e., 830 nm, the real-time wavelength shift was obtained. With this experimental procedure, the kinetic monitoring of sucrose hydrolysis was realized.

**Figure 6:**
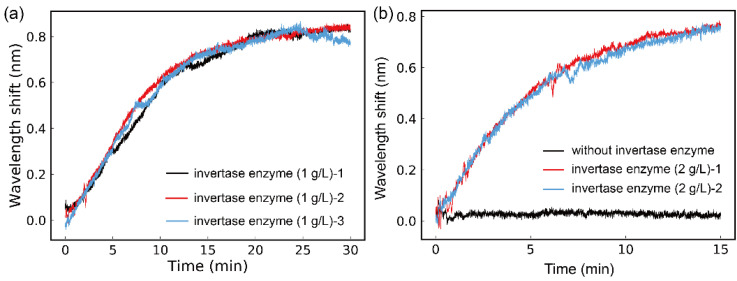
(**a**) The monitoring of the hydrolysis with 1 g/L of invertase enzyme. (**b**) Two hydrolysis measurements with 2 g/L of invertase enzyme and the measurement with not invertase enzyme.

Firstly, the measurements under 1 g/L of invertase enzyme was implemented. Through the kinetic monitoring, the real-time reaction state in the SC was observed intuitively as Figure 6 presents. With the same experimental procedures, the hydrolysis with 1 g/L of invertase enzyme was monitored for 3 times, and the results are shown in Figure 6a For a reference, the sucrose in SC without invertase enzyme was also detected as displayed in Figure 6b.

Figure 6a depicts the repeatability of this method via multiple measurements, which were carried out with the invertase enzyme of 1 g/L. It means that the monitoring of the hydrolysis reaction with this weak measurement system is reasonable and reliable. Also, as shown in Figure 6b, with this monitoring technology, it could be clearly noticed that the invertase enzyme played a vital catalytic role in sucrose hydrolysis. Under the action of invertase enzyme with a concentration of 2g/L, the hydrolysis performed rapidly until the reaction was finished complementally. Rather, without the participation of invertase enzyme, as the black line in Figure 6b exhibited, the hydrolysis of sucrose hardly generated in a short period of time. Such a comparison provided a further evidence for the feasibility and reliability of this weak measurement scheme for the kinetic study of the chiral reaction. As Figure 6 revealed, some unpredictable fluctuations, which might be caused by the inevitably nonuniform distribution of the chiral molecule in the SC, appeared in the real-time monitoring process. Nevertheless, the general trend of the curves was reasonable and reliable for the kinetic studies of sucrose hydrolysis. And the repeatability was clearly reflected from Figure 6a,b.

### 3.4. Real-Time Monitoring of Sucrose Hydrolysis under Different Catalytic Conditions

**Figure 7:**
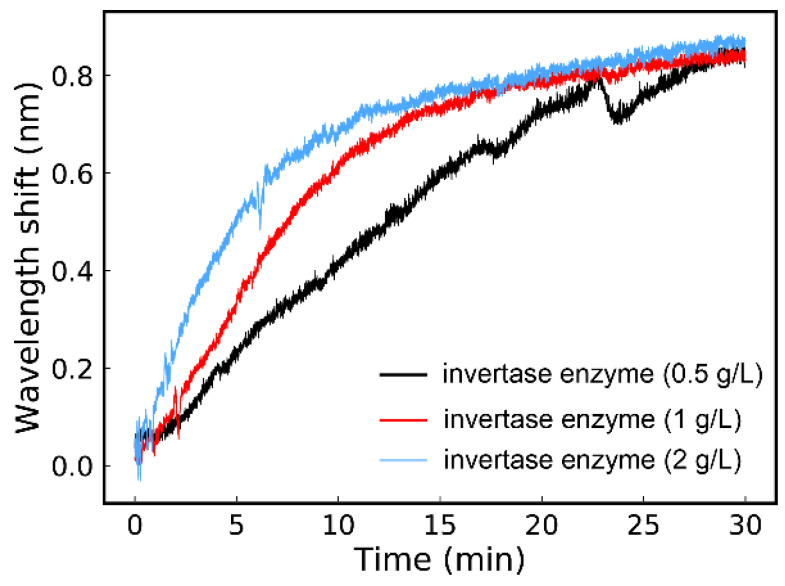
The real-time response of the wavelength shift for sucrose hydrolysis with different invertase enzymes.

Here, 2 mL of invertase enzyme with different concentration including 0.5 g/L, 1 g/L and 2 g/L was utilized for the catalyzing of sucrose hydrolysis, respectively. The real-time hydrolysis curves of the hydrolysis in varied situations were achieved in Figure 7.

As described in Equation (3) and Figure 5, in this weak measurement system, the wavelength shift of output spectrum was related to the specific optical rotation of the chiral sample in SC. For the reaction of sucrose hydrolysis, with the action of invertase enzyme, sucrose converted to equimolar mixture of fructose and glucose. Simultaneously, the optical rotation of the solution changed as the reaction progress. Since specific optical rotation of sucrose, glucose and fructose was 66.6°, 52.5° and −92.4° respectively, the whole specific optical rotation of the reaction solution would transform from a positive angle to a negative angle. According to the exploration of Figure 5, the sign of the optical rotation angle to the wavelength shift showed opposite influence on the wavelength shift. Figure 5 indicated that the wavelength shift had a positive correlation with increasing concentration of fructose. In the hydrolysis reaction, the concentration of glucose and fructose increased with the decrease of sucrose concentration. Thus, the wavelength would shift with a positive tendency, which is consistent with the experimental results shown in Figure 6 and Figure 7. As Figure 7 displayed, there was a rapid jump on the black curve, which was caused by a tiny bubble occasionally produced in the hydrolysis processing. It neatly illustrated the necessity of real-time detection, which could tell us what was happening in the chemical reaction.

The experimental curves in Figure 7 exhibited the kinetic response relation of the hydrolysis reaction. The slope of the curve represented the reaction speed of the hydrolysis process. According to the tendency of the real-time hydrolysis curve, in the initial reaction period, the sucrose hydrolysis rapidly. Then, the enhancement of the wavelength shift got saturated with gradual completion of the reaction. Obviously, different concentrations of invertase enzyme have great influence on the reaction rate. In these three situations, the higher the concentration of the invertase enzyme is, the faster saturation point reached. Despite the reaction rates are different, the amount of product is virtually the same at the end of the hydrolysis.

**Figure 8:**
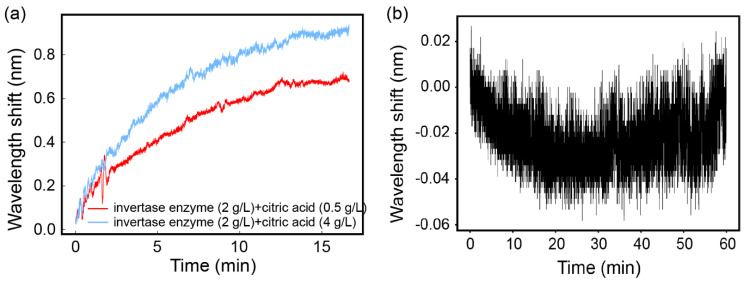
(**a**) The hydrolysis with invertase enzyme and citrate. (**b**) The blank measurement for deionized water in 1 h.

For a further investigation of sucrose hydrolysis in different circumstances, citrate was exploited in the reaction. The citrate with the concentration of 0.5 g/L and 4 g/L were prepared for this experiment. The sucrose hydrolysis was carried out with the combined effect of the invertase enzyme and citrate. The real-time monitoring of the hydrolysis process in the two conditions were represented with Figure 8a. Apparently, the citrate promoted the hydrolysis reaction. The reaction rate increased with higher concentration of citrate. Moreover, according to the amount of wavelength shift, in the situation with 4 g/L of citrate, the optical rotation of the solution is much higher than that with another concentration. That may result from the fact that sucrose hydrolysis was more thorough under the acidic conditions. The blank measurement shown in Figure 8b was realized by taking deionized water as the sample and monitoring the wavelength shift for 1 h. It reveals the stability of the system in a certain condition, including a stable environmental temperature.

Sucrose hydrolysis is a typical first order reaction, the reaction rate is proportional to the logarithm of the concentration:

(5) $$\mathrm{lnc}=-kt+\ln c_{0}$$

where $c_{0}$ is the initial concentration of sucrose, while c is the concentration of sucrose over the changing time t. The real-time concentration of sucrose is calculated by Equation (6):

(6) $$C_{1}=e^{-kt+\ln C_{0}}=C_{0}e^{-kt}$$

Here, $c_{1}$ is the concentration of sucrose at the timing point of t. With the relationship described in Equation (4), the rotation angle α of sucrose can be expressed with $\;\alpha_{1}$, as shown in Equation (7):

(7) $$\alpha_{1}=\frac{\left[\alpha \right]\cdot l\cdot c_{1}}{100}=\frac{\left[\alpha_{1}\right]\cdot l\cdot c_{0}e^{-kt}}{100}$$

For the chemical reaction exhibited in Figure 3, the concentrations of glucose and fructose, which were expressed as $c_{2}$ and $c_{3}$, can be calculated with initial and real-time concentration of sucrose. Then, the rotation angle of them, which are defined as $\;\alpha_{2}$ and $\alpha_{3}$ respectively, can be correspondingly obtained:

(8) $$c_{2}=c_{3}=\frac{10}{19}\left(c_{0}-c_{1}\right)\;\alpha_{2}=\frac{\left[\alpha_{2}\right]\cdot l\cdot \left(c_{0}-c_{1}\right)}{190}\;\alpha_{3}=\frac{\left[\alpha_{3}\right]\cdot l\cdot \left(c_{0}-c_{1}\right)}{190}$$

Hence, the total rotation angle α of the solution can be derived with Equation (9):

(9) $$\alpha =\alpha_{1}+\alpha_{2}+a_{3}=\frac{l}{100}\left[\alpha_{1}\right]\cdot c_{0}e^{-kt}+\frac{l\cdot c_{0}}{190}\left(1-e^{-kt}\right)\cdot (\left[\alpha_{2}\right]+\left[\alpha_{3}\right]$$

According to Equations (3) and (9), the reaction rate v can be acquired by taking the derivative, as shown in Equation (10):

(10) $$v=\frac{d\left(\delta \lambda \right)}{dt}=-\frac{2\pi {\left(\Delta \lambda \right)}^{2}}{\lambda_{0}}\sec^{2}(\frac{\pi }{4}-\frac{\beta }{2}-\alpha )\left(-\frac{d\alpha }{dt}\right)\;=\frac{2\pi {\left(\Delta \lambda \right)}^{2}}{\lambda_{0}}\sec^{2}(\frac{\pi }{4}-\frac{\beta }{2}-\alpha )\cdot \frac{k\cdot e^{-kt}\cdot l\cdot c_{0}}{10}\left(\frac{\left[\alpha_{2}\right]+\left[\alpha_{3}\right]}{19}-\frac{\left[\alpha_{1}\right]}{10}\right)\;=\frac{\pi {\left(\Delta \lambda \right)}^{2}}{5\lambda_{0}}\cdot \;ke^{-kt}\cdot l\cdot c_{0}\left(\frac{\left[\alpha_{2}\right]+\left[\alpha_{3}\right]}{19}-\frac{\left[\alpha_{1}\right]}{10}\right)\;\sec^{2}(\frac{\pi }{4}-\frac{\beta }{2}-\alpha )$$

**Figure 9:**
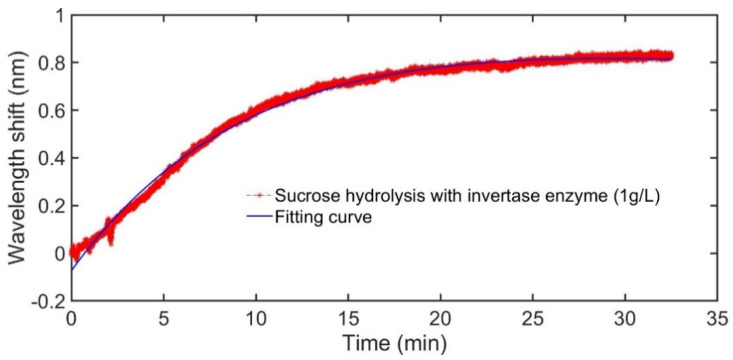
The numerical fitting of experimental data.

To further obtain the sucrose hydrolysis rate, we performed exponential fitting with the experimental result of sucrose hydrolysis, which was shown in the red curve in Figure 7. The fitting result is shown in Figure 9. The reaction rate can be obtained by taking the derivation of the fitted curve:

(11) $$v=\frac{d\left(\delta \lambda \right)}{dt}=0.0081e^{-0.0074t}+0.12e^{-0.098t}$$

Within a certain time range, the reaction rate expressed by Equation (11) can be approximately regarded as equal to $0.12e^{-0.098t}$. According to Equations (10) and (11), a reaction rate constant of 0.098 can be derived under this experimental condition. A comprehensive dynamic analysis of the sucrose hydrolysis is thus realized with this weak measurement system.

**Figure 10:**
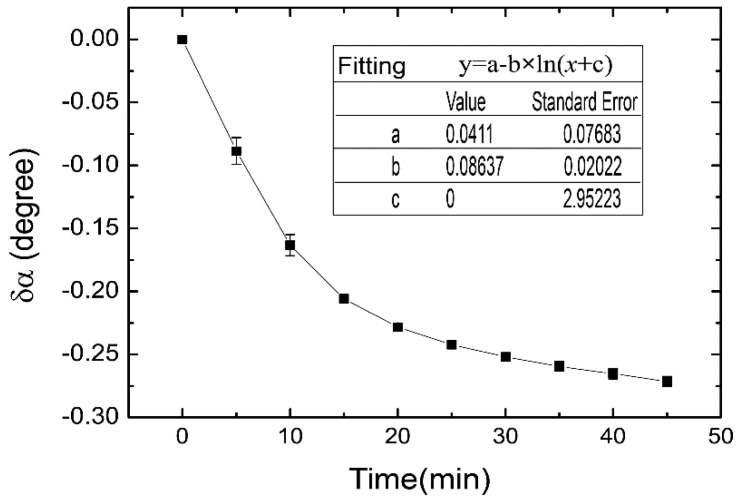
Optical rotation detection for the sucrose hydrolysis with commercial polarimeter. The error bar was obtained with three measurements.

As a comparison, the measurement of the sucrose hydrolysis was implemented with a traditional commercial polarimeter (JASCO, P-1010), which was provided by Pharmacy School of Zhejiang University. In the same experimental condition for Figure 9, which was accomplished with weak measurement, the sucrose (10 g/L) with a volume of 12 mL was catalyzed by 2 mL of invertase enzyme (1 g/L). The experimental result was exhibited in Figure 10.

In this polarimeter, the sampling time is 1s, and the wavelength of work light was 589 nm. The optical rotation angle change of the reaction solution, i.e., the parameter of δα in Figure 10, was detected every five minutes. The fitted resulted was displayed in the inset. It reveals that the hydrolysis speed rate, i.e., the fitting parameter of b, should be 0.086, which was a little smaller than that obtained with weak measurement. It was because the experimental temperature of this experiment, which was about 18 °C, was lower than that of the work accomplished in summer. Hence, such an investigation also innovated the reliability of the chiral sensor based on weak measurement in real-time monitoring of chemical reaction.

## 4. Conclusions

An optical rotation detection system based on weak measurement was built for the study of chiral analysis, especially for the monitoring of sucrose hydrolysis. The optical rotation of chiral sample provided a modification to the preselection polarization of the weak measurement system. Due to the weak value amplification in the frequency domain for optical rotation detection, the polarization angle corresponding to the optical activity of chiral molecule could be determined by the central wavelength shift of output spectrum. With this method, the optical rotation of sucrose, glucose and fructose was detected. Also, by fitting the central wavelength of output spectrum in real time, we could monitor the varied chirality of the sample. In this work, this system was utilized in the kinetic study of sucrose hydrolysis, which produces an equimolar mixture of glucose and fructose. The specific optical rotation of sucrose, glucose and fructose have a great distinction about the absolute value as well as the sign. Hence, the optical rotation of the solution, which was monitored with the wavelength shift, would change as the reaction progressed. In this work, the hydrolysis process was implemented with the action of invertase enzyme. The real-time spectrum analysis with LabVIEW provided the real-time hydrolysis curve for the hydrolysis process. With such a kinetic monitoring, the reaction rate as well as the extent was intuitively displayed. The repeatability and reliability were demonstrated by multiple measurements and the compared experiments, which included the hydrolysis process with invertase enzyme or not. Meanwhile, the higher concentration invertase enzyme had better catalytic effect on sucrose hydrolysis, while the amount of the final products just relied on the initial level of the sucrose. In addition, the investigation about the influence of citrate to the sucrose hydrolysis was implemented. Results showed that the citrate further promoted the catalyzing of the hydrolysis reaction on the basis of invertase enzyme. Combining with theoretical analysis and numerical fitting, the reaction rate was achieved. By performing a comparative experiment with a standard polarimeter, the feasibility and reliability of this weak measurement system for real-time chiral analysis was demonstrated. In conclusion, our proposed chiral sensor based on weak measurement fulfilled its potential in chiral analysis, especially in the kinetic study of the chiral reaction. It was critical for the chiral research in real time, such as food science, pharmaceutical synthesis and analysis.

## Data Availability

Not applicable.
